# Early Forest Fire Detection Using Radio-Acoustic Sounding System

**DOI:** 10.3390/s90301485

**Published:** 2009-03-03

**Authors:** Yasar Guneri Sahin, Turker Ince

**Affiliations:** 1 Izmir University of Economics, Department of Software Engineering, Sakarya Cad. No:156, Balcova/Izmir, Turkey; E-Mail: yasar.sahin@ieu.edu.tr; 2 Izmir University of Economics, Department of Computer Engineering, Sakarya Cad. No:156, Balcova/Izmir, Turkey; E-Mail: turker.ince@ieu.edu.tr

**Keywords:** Forest fire detection, RASS, acoustic sounding, radar, emergency management

## Abstract

Automated early fire detection systems have recently received a significant amount of attention due to their importance in protecting the global environment. Some emergent technologies such as ground-based, satellite-based remote sensing and distributed sensor networks systems have been used to detect forest fires in the early stages. In this study, a radio-acoustic sounding system with fine space and time resolution capabilities for continuous monitoring and early detection of forest fires is proposed. Simulations show that remote thermal mapping of a particular forest region by the proposed system could be a potential solution to the problem of early detection of forest fires.

## 1. Introduction

Forest fires are one of the most important and prevalent type of disasters and they can create great environmental problems for Nature. It is known that they are detectable and easily preventable. When a wildfire burns out of control, the size of the losses can be almost immeasurable. The cost of such disaster may be millions of trees, in addition to losses of structures, animals (wild and farm), and human life.

One and possibly the most important method for protecting forests from wildfires is their early detection. The earliest possible detection enables a rapid response to minimize the spread. Moreover, information regarding the seat of the fire is invaluable for the rapid deployment of fire-fighters. Therefore, early detection, containment at the early stages and extinguishment of a fire before it spreads are crucial for wildfire management.

A number of early forest fire detection methods have been proposed using various remote sensing systems based on infrared thermal camera (Therma-cam) imaging, airborne or ground-based Lidar and Satellite-based Synthetic Aperture Radar (SAR) imaging techniques [1–3]. In this paper, an early detection system for crown and surface wildfires based on radio-acoustic sounding (RASS) system allowing remote temperature measurements and thermal sensing of a particular forest region. As opposed to some of the aforementioned remote sensing systems that depend on smoke plume detection, the RASS technique with its high resolution volume, can directly and continuously measure air temperature profiles, including temperature increase due to fire.

## 2. Related Works and Motivation

Recently, many different forest fire detection and fire management system have been developed and successfully applied. It is fact that forest fire detection and forest fire management should be distinguished to understand how to setup new systems for fire detection and containment. Hence, we divided forest fire detection and management systems into different classes.

A great deal of research into forest fire management has been published in recent years, presenting a variety of solutions and management techniques. Some research has combined forest fire management and detection methods [4–6]. One group of researchers has studied forest fire prevention through an analysis of cause [7–11]. In addition, other studies have dealt with private management systems for particular forest areas. Although, some forest fire management systems include forest fire detection systems, containment and extinguishment, etc, detection is not their essential focus, hence, fire detection systems should be considered alone for establishing more accurate detection systems.

Forest fire detection systems can, in fact, be divided into three main categories: image processing based systems, thermal based systems and other applications. At this point, the first category (image processing based systems) can be further divided into two categories: one based on satellite images and another based on fixed camera shoots. Similarly, thermal based systems can also be divided into two different categories: static and dynamic sensor based, and remote sensing based. The latter uses tools such as Radar, Lidar (Light Detection and Ranging), and Sodar (Sound Detection and Ranging).

In literature, many authors focus on early fire detection by means of image processing techniques on satellite images gathered from forests [12–27]. Although all are useful scientific studies and a number have been successfully applied, satellite based systems studies have limitations, including testing limited to private forest areas, high costs and the requirement of a suitable satellite. Many other studies can be found in the forest fire detection literature using fixed camera, infrared and thermal images [28–35]. Furthermore, some researches focus particularly on LIDAR systems [36–42].

Although all these explorations were successful, because of their nature, usage of these systems without support from other assistive systems is insufficient. Since thermal cameras are ineffective in daytime in summer, fixed cameras are ineffective in dark weather (evening, cloudy, night etc.) and infrared cameras cannot detect smoke, they must be supported by other systems. Where thermal sensors are used, they are attached to fixed coordinates, restricting the detection area and therefore requiring many sensors to cover a single hectare of forest.

Different techniques have been proposed for fire detection and fire management. Some have been developed to reduce single system disadvantages in the area of fire detection while others have proposed extraordinary solutions to fire detection. A good example is the study of Alexander *et al*., which focused on role of composting in forests for fire detection and prevention [43]. Another early study focused on the use of decision making techniques in building a centralized forest management system [44]. Sahin addressed the role of animals as biological sensors in forest fire detection [45]. Other research has presented a method for early forest fire detection in hilly terrains [46]. In addition, a considerable amount of further fire management research has been carried out [47–49]. The application of emergent high technology to fire management, using state of the art detection systems has resulted in the achievement of more accurate results.

This study presents a proposal for using radio-acoustic sounding to create thermal maps of forest areas for detection of potential fires. The main basis is the fact that the radio-acoustic sounding technique has much higher sensitivity to temperature changes and can remotely provide more accurate air temperature measurement, crucial to early detection, than any of the other aforementioned remote sensing systems. Additionally, the proposed system, which is able to continuously monitor simultaneous multiple ranges with fine spatial and temporal resolution, is a more efficient and cost effective solution than a distributed network of multiple static sensors.

## 3. Temperature Measurement by Radio-Acoustic Sounding

The concept of using wave radiation to infer atmospheric structure and parameters has been applied for decades. Scientists have recognized that sound waves are particularly sensitivity to temperature and wind, which can be transformed into the inverse problem of obtaining information about atmospheric temperature and wind velocity fields [50]. Therefore, acoustic parameters characterizing sound propagation through the atmosphere, such as sound velocity, can carry information providing a spatial description of atmospheric properties such as virtual air temperature and fluid motion (wind or turbulence). The effective velocity of an acoustic ray propagating through the atmosphere is given by

(1) $${\vec{c}}_{\mathrm{eff}}=c\vec{n}+\vec{v}$$

(2) $$c=20.05\theta_{v}^{1/2}=20.05\sqrt{\theta (1+0.61q)}$$

where *n⃗* is the unit wave front normal, *v⃗* is the wind velocity, and c is the sound speed which is *θ_v_* being the virtual temperature, *θ* the potential temperature, and q the mixing ratio. According to equations (1) and (2), the travel time of sound in air is mainly affected by the distribution of the temperature and by the motion of the fluid along the sound wave path. Therefore, experimental acoustic backscatter data will result from superposition of these different types of data, and an accurate estimation of the fluid field motion is necessary for distinguishing between the scalar influence of the temperature and the influence of the vector fluid.

Sodar (SOnic Detection And Ranging), or acoustic radar, operates by transmitting acoustic pulses into the atmosphere and detecting echoes backscattered from mainly atmospheric thermal inhomogeneities [51]. Several researchers have designed and presented systems using sound signals in the audio and near-audio frequency range to characterize meteorological flow and heating phenomena from near-surface layers to a height of several kilometers in the atmosphere [52, 53]. However, due to the inability of monostatic Sodar to directly measuring temperature, a radio-acoustic sounding technique has been developed as a reliable tool for remotely measuring virtual temperature *θ_v_*. It has been both theoretically and experimentally demonstrated that the two or three dimensional distributions of temperature and flow fields can be remotely measured and estimated by bistatic acoustic or radio-acoustic remote sensing systems employing different techniques and algorithms. Radio-acoustic sounding system (RASS) is capable of measuring virtual temperature profiles (with range) using a Doppler radar to measure the propagation speed of a sound wave, which is directly related to the square root of virtual temperature [54].

According to RASS theory, acoustic waves are transmitted into the atmosphere at approximately half the wavelength of the radar (Bragg matching condition) and the speed can then be detected and calculated by the Doppler effect at the radar receiver provided that the acoustic and radar beams overlap. The method relies on the enhancement in electromagnetic backscatter due to the Bragg resonant scattering. In practice, a range of acoustic frequencies must be used to satisfy the Bragg matching condition for the range of expected speeds of sound since it varies due to temperature and wind variations with range. The temperature and wind vector field can thus be determined from analysis of the intensity and the Doppler frequency shift of the return signal. Therefore, the operating frequency and acoustic power transmitted into the atmosphere affect performance of a RASS system through the frequency dependent attenuation of sound by the atmosphere [55]. It is worth noting that the capability of the Doppler radar to measure wind speed and direction is vital to retrieval of the true speed of the sound since it must be subtracted from the Doppler velocity measured by the radar.

**Figure 1.**
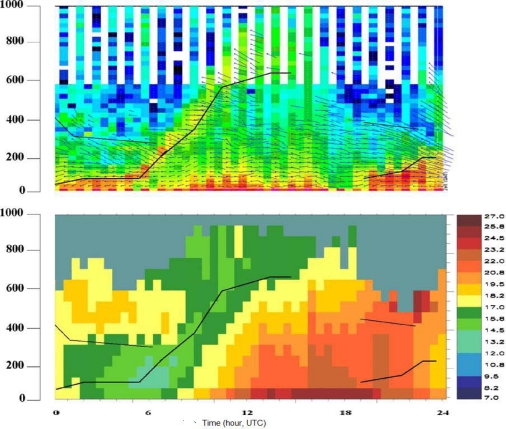
Top frame: Sample RASS backscatter intensity and horizontal winds profiles. Bottom frame: The estimated temperature profile in degrees Celsius [58].

In this study we propose to utilize the radio-acoustic remote sensing technique described above to monitor the atmospheric temperature in forest areas. Specifically, by remote measurement of temperature variations using a RASS, continuous thermal characterization of a forest area would be possible allowing near real-time fire detection and characterization. Additionally, wind vector field measurement could provide additional information about the propagation path of a possible fire. It is also possible to combine the proposed radio-acoustic sensor with an optical sensor, such as Lidar, which is more sensitive to smoke plumes or aerosol content (rather than thermal structure) since backscattering is dominated by solid or liquid particles at the size of the wavelength of light. Thus, the fusion of data from radio-acoustic and optical sensors could provide more information depending on sensitivity and resolution of multiple sensors. Acoustic Sodar systems have been designed in the past with acoustic excitation (at transmitter) that is either pulsed, phase or frequency-coded, such as frequency-modulated continuous-wave (FMCW). Angevine *et al*. showed that a frequency modulated acoustic signal of constant amplitude gives the best performance [56]. For the radar system, the relatively simple, low cost FMCW Doppler radar with two bistatic antenna can provide high average transmit power and fine range gate resolution. For FMCW type source excitation, the receiver could simultaneously detect backscatter intensities from different targets in the operating range using standard signal processing techniques. In this case, signal processing of the echo usually involves matched filtering, which is most commonly implemented for all ranges simultaneously through spectral analysis via an FFT algorithm [57]. In environmental monitoring applications, the change detection and identification, such as temperature increase due to fire, can be achieved by measuring differential backscatter intensity profiles and applying statistical hypothesis testing methods. Figure 1 illustrates sample RASS measured backscatter intensity and horizontal wind profiles as well as the estimated temperature profile in degree Celsius from [58].

## 4. Proposed System

**Figure 2.**
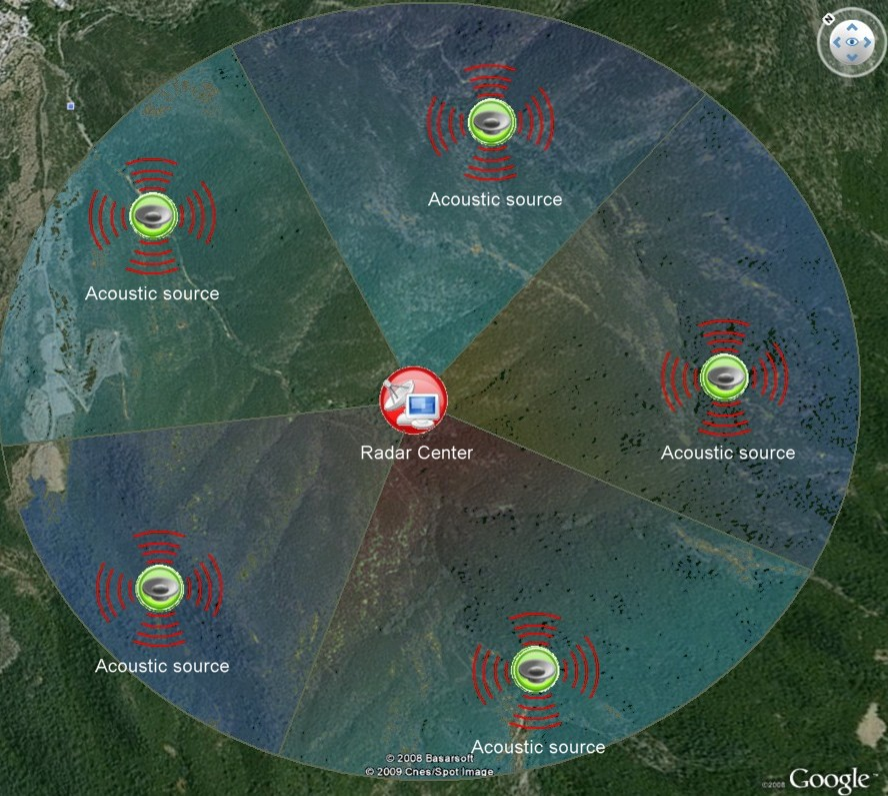
Proposed system infrastructure.

The system is simply based on the rule that warm air rises rapidly, and if a temperature map of the air immediately above the trees is periodically created, sudden temperature changes can be immediately identified. The proposed system infrastructure is shown Figure 2.

The system consists of two main device types: Radars with fire watchtower, Acoustic sources. While acoustic sources generate sound waves with a certain frequency and a certain power level, Radar continuously scans for acoustic waves. As is known, sound wave speed is affected by air temperature. Radar can scan the differences in speed of acoustic waves, generated by acoustic sources, periodically measuring air temperatures immediately above the trees, over an area of 12–28 km^2^ area (depending on placement of the acoustic sources). Software embedded in Radar creates a thermal map of the region and stores thermal data for future use. Then the software checks for instant changes in temperature, and when sudden increases are found, establishes communication with the relevant fire watchtower and warns staff of the location.

Acoustic sources are placed in the particular forest regions (especially, on high ground points that adequately overlook the circular region to a radius of 2–3 km). In order to prevent problematic echoes in the collected data, problems that appear to be caused by ground clutter are identified and recorded after the acoustic sources are installed in the circular regions. Occasionally the location of an acoustic source needs to be adjusted until the optimum location is achieved.

Two restrictions and constraints must be considered at this point, these are the scanning area of the Radar, and frequency and the power level of acoustic sources. The scanning area of Radar should be restricted to a certain level of altitude immediately above the trees. In addition, the determination of the frequency and power of the acoustic sources should take into careful consideration factors such as forest surface and density, and distance between Radar and acoustic sources, etc.

The frequencies of a (acoustic sources) and r (Radar) should be proportional according to equation 1. Moreover, the power level of acoustic sources is directly related with the coverage area and there is a correlation between the frequency and the power level of the sound waves. Table 1 shows possible constraints on frequencies and power level of acoustic sources.

As can be seen in the Table, in order to reduce the effect of high volume sound on the forest environment, sound frequency should be set as high as possible, although this involves higher levels of power use.

### 4.1. Operational Details and Simulation Results

**Figure 3.**
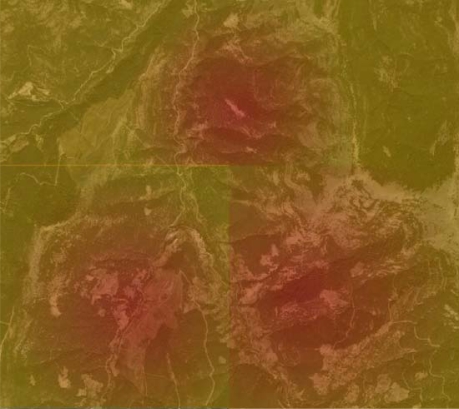
Simulated thermal map of a forest

After the installation, system operates as follows: first, the Radar measures the temperature at multiple distances using acoustic signals, and the data is determined in 5 m increments over a circular area, from 10 to 2,000–2,500 m (from the acoustic source), giving an effective panning area of approximately 19.6 km^2^. After gathering the thermal data from the forest, a rough thermal map is created. A simulated rough thermal map is shown in Figure 3.

**Figure 4.**
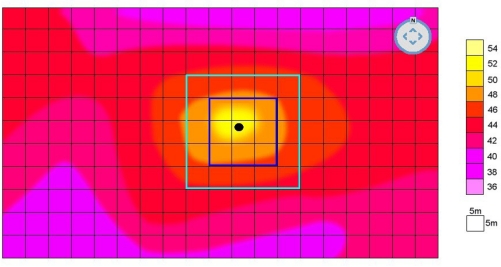
Refined thermal map of a warm region.

The system begins to monitor the area, comparing temperature anomalies to seasonal thermal norms, which were previously stored in the system. The size of anomalies varies according to area structure, in the simulation, an increase of 7 °C was chosen as the anomaly. When an anomaly is detected in a certain area, the system makes related Radar focus on that area to create a refined thermal map. A simulated detailed thermal map of a certain area is shown in Figure 4.

**Figure 5.**
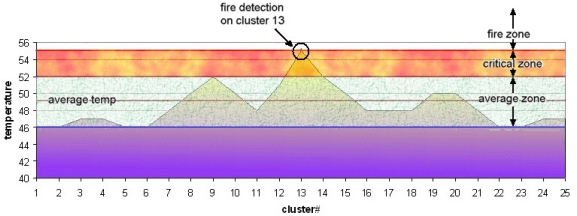
Warm region thermal data spectrum.

A refined thermal map is created for a specified area and divided in to clusters (in simulation that cluster size was selected 5×5 m = 25 m^2^). Following this, the exact point of the thermal anomaly is detected and identified. The temperature is compared with the selected level two neighbor clusters (in simulation, 8 clusters for 1st level, 16 clusters for 2nd level). Finally a spectrum graph showing the temperature measurements for specified point and its neighbors is sketched. Figure 5 shows such a graph for the simulated data.

**Figure 6.**
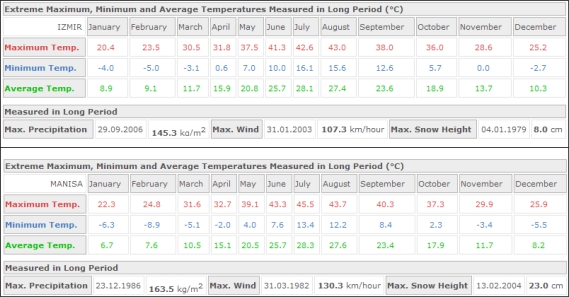
Seasonal weather data for Izmir and Manisa [59,60]

There are three different thermal zones shown in the graph, which can be employed in two different fire detection methods. In the first method, possibility of a fire is indicated by existence of a temperature value in a fire zone when the graph is checked. In the second, the top temperature is found and compared to temperatures in neighboring clusters. A steep increase or 6–10 °C higher than average temperature indicates possible fire. Consequently, thermal anomaly data is sent to the fire watchtower and location indicated is checked. Table 2 shows all the simulation environment data and the assumptions, and Figure 6 shows seasonal weather data for Izmir and Manisa. Simulation assumptions were based on the seasonal weather data for the specified areas.

Five acoustic sources and a single watchtower with radar were defined the simulation, which established a panning area of approximately 98 km^2^ (19.2 km^2^ for each acoustic source). 55 °C was determined as the upper limit for the critical zone, and temperatures above this were in fire boundary, according to seasonal thermal norms of selected forest area (Izmir - Manisa). The simulation results showed that RASS can supply data with as much as 80% accuracy in crown and surface fires. According to simulation results, the proposed system, however, could not give reasonable results for ground fires because of spread behavior.

### 4.2. Effectiveness and Economical View of the Forest Fire Detection System

In general, fire detection systems such as Lidar, RASS, IR and Image processing based on lookout towers are more expensive than detection systems based on observation towers since RASS, Lidar etc. require the use of emergent technologies and some extra equipments and devices.

In fact, measures of detection system effectiveness and total cost depend on many relative parameters. The calculation of total cost and system effectiveness are, hence, relative. Marteel and McAlpine set some measurement aspects of detection systems’ cost and effectiveness such as “*Cost per unit area protected”, “Cost per fire detected”, “Hours flown per fire detected”, “Percent of fires detected by airborne observers (compete with the public)”, “Average size at detection (ignores travel time, spread rate, etc.)”* [61].

Additionally, some other parameters must also be included to this list such as energy consumption, sustainability of the system, cost of sustain the system, etc. As stated in the introduction section, when a wildfire burns out of control, the size of the losses can be almost immeasurable since a disastrous fire can cost millions of trees, as well as structures, animals, and human life, the cost of fire may, therefore, be too high.

Consequently, it is very difficult to determine which system is more efficient and more effective in forest fire detection. Structure of the forests, government strategies about fire management, current technology availabilities must also be considered in deciding which type of forest fire detection system is more appropriate for specific use. As a result, we propose a system that might save millions of tree from wildfires.

## 5. Conclusions

In this paper we proposed a method which uses RASS signals to remotely obtain thermal maps of the forest and allows early detection of fires using these thermal maps. The results gathered from the simulation showed that the proposed system is suitable for crown and surface but not ground fires which can be investigated in future work. In addition, this system can be combined with existing systems to augment the efficiency of fire detection for all types of wildfires. In conclusion, jungles and forests on the Earth are vanishing dramatically due to man-caused fires and various other reasons, life is, hence, becoming more critical for all creatures. This paper proposes a novel method of early stage fire detection.

## Figures and Tables

**Table 1.** Possible frequencies and power level of acoustic sources and Radar.

$f_{a}=\frac{C_{a}}{\lambda_{a}}$**(KHz)**	$f_{r}=\frac{C_{r}}{\lambda_{r}}$**(MHz)**	**Pr (W)**	**Pa (W)**	**Range coverage (radius)**

1.035	457.5	30	18	200 m – 2.5 km
2.070	915.0	35	20	200 m – 2.5 km
3.105	1372.5	40	30	200 m – 2.5 km
4.140	1830.0	45	40	200 m – 2.5 km
8.280	3660.0	55	80	200 m – 2.5 km
16.560	7320.0	70	160	200 m – 2.5 km

**Table 2.** Simulation environment data

Subject	Data/Assumption/Value
Place	Manisa-Izmir – Turkey
Season	Summer (July–August)
Upper limit for critical zone	55°C (because the seasonal norms shows that the maximum temperature can be 54°C for that area under sunlight) [59,60]
Number of sound sources	5 (each source can be effective for 19.6 km^2^)
Approximate coverage area	98 km^2^ (5 acoustic sources × 19.6 km^2^ for each acoustic source)
*f_a_*	8.280 KHz
*f_r_*	3,660 MHz
Pr	55 W
Pa	80 W
Threshold temp value for anomaly	7°C (this value (6°C–10°C) obtained using the air temperature difference between sunlight and shade for the specified forest area) [59,60]
Critical zone temp	52°C [59,60]
